# The Turkish COVID-19 Anxiety Syndrome Scale: Psychometric validation and association with psychological symptoms

**DOI:** 10.1192/j.eurpsy.2023.1670

**Published:** 2023-07-19

**Authors:** F. Obuca, A. Bilge, P. Ünal-Aydın, O. Aydın, D. C. Kolubinski, M. M. Spada

**Affiliations:** 1 Department of Psychology, International University of Sarajevo, Sarajevo, Bosnia and Herzegovina; 2 Department of Behavior, Neurobiology and Cognition, University of Vienna, Vienna, Austria; 3 School of Applied Sciences, London South Bank University, London, United Kingdom

## Abstract

**Introduction:**

The COVID-19 Anxiety Syndrome Scale (C-19ASS) is a reliable and valid tool developed for assessing unhealthy coping strategies that arise due to fear and threat of COVID-19.

**Objectives:**

The main aim of this study was to validate the Turkish version of the C-19ASS. Additionally, we explored the relationship between COVID-19 anxiety syndrome, personality traits, and psychological variables.

**Methods:**

We recruited 296 Turkish adults using the convenience sampling method. Participants completed the Turkish version of the C-19ASS and a battery of measures assessing COVID-19 anxiety, health anxiety, well-being, personality traits, and social adaptation. A Confirmatory Factor Analysis (CFA) was conducted on the C-19ASS, and validity was evaluated by analyzing correlations with other instruments.

**Results:**

The results of CFA have confirmed a 9-item two-factor structure. The Turkish version of the C-19ASS showed adequate internal consistency, convergent validity, and incremental validity. Hierarchical regression analysis revealed that C-19ASS Perseveration is a significant negative predictor of well-being when controlling for age, COVID-19 anxiety, health anxiety, personality traits, and social adaptation.

**Conclusions:**

The Turkish version of the C-19ASS was found to be a reliable and valid measure of the COVID-19 anxiety syndrome. C19-ASS may be a helpful follow-up tool for examining specific psychological symptoms of the COVID-19 pandemic.

**Disclosure of Interest:**

None Declared

